# Health service utilisation for acute respiratory infections in infants graduating from the neonatal intensive care unit: a population-based cohort study

**DOI:** 10.1186/s12887-023-04152-5

**Published:** 2023-07-01

**Authors:** Paul G. Stevenson, Matthew N. Cooper, Wesley Billingham, Nicholas de Klerk, Shannon J. Simpson, Tobias Strunk, Hannah C. Moore

**Affiliations:** 1 Telethon Kids Institute, The University of Western Australia, Perth, WA Australia; 2 Wal-yan Respiratory Centre, Telethon Kids Institute, Perth, WA Australia; 3 School of Allied Health, Curtin University, Perth, WA Australia; 4 Neonatal Directorate, Child and Adolescent Health Service, Perth, WA Australia; 5 Wesfarmers Centre of Vaccines and Infectious Diseases, Telethon Kids Institute, PO Box 855, West Perth, WA 6872 Australia; 6 School of Population Health, Curtin University, Perth, WA Australia

**Keywords:** Acute respiratory infection, Neonatal intensive care unit, Hospital morbidity, Record linkage, Infant, Child, Preterm

## Abstract

**Background:**

Despite advances in neonatal intensive care, babies admitted to Neonatal Intensive Care Units (NICU) suffer from adverse outcomes. We aim to describe the longer-term respiratory infectious morbidity of infants discharged from NICU using state-wide population-based linked data in Western Australia.

**Study design:**

We used probabilistically linked population-based administrative data to analyse respiratory infection morbidity in a cohort of 23,784 infants admitted to the sole tertiary NICU, born 2002–2013 with follow up to 2015. We analysed incidence rates of secondary care episodes (emergency department presentations and hospitalisations) by acute respiratory infection (ARI) diagnosis, age, gestational age and presence of chronic lung disease (CLD). Poisson regression was used to investigate the differences in rates of ARI hospital admission between gestational age groups and those with CLD, after adjusting for age at hospital admission.

**Results:**

From 177,367 child-years at risk (i.e., time that a child could experience an ARI outcome), the overall ARI hospitalisation rate for infants and children aged 0–8 years was 71.4/1000 (95% confidence interval, CI: 70.1, 72.6), with the highest rates in infants aged 0–5 months (242.9/1000). For ARI presentations to emergency departments, equivalent rates were 114/1000 (95% CI: 112.4, 115.5) and 337.6/1000, respectively. Bronchiolitis was the most common diagnosis among both types of secondary care, followed by upper respiratory tract infections. Extremely preterm infants (< 28 weeks gestation at birth) were 6.5 (95% CI: 6.0, 7.0) times more likely and those with CLD were 5.0 (95% CI: 4.7, 5.4) times more likely to be subsequently admitted for ARI than those in NICU who were not preterm or had CLD after adjusting for age at hospital admission.

**Conclusions:**

There is an ongoing burden of ARI in children who graduate from the NICU, especially those born extremely preterm, that persists into early childhood. Early life interventions to prevent respiratory infections in these children and understanding the lifelong impact of early ARI on later lung health are urgent priorities.

**Supplementary Information:**

The online version contains supplementary material available at 10.1186/s12887-023-04152-5.

## Background

Improvements in obstetric care and neonatal practice have led to significant reductions of neonatal mortality in high-income settings [1–3]. However, over the past decade, studies utilising large population cohorts have reported variable admission rates to Neonatal Intensive Care Units (NICUs) including increasing rates [4] and decreasing rates [5]. Furthermore, preterm birth, one of the more common reasons for NICU admission, remains a substantial health problem on a global level [6] and the prevalence of chronic lung disease of prematurity (CLD) has not changed [7].

Globally, acute respiratory infections (ARI) are a leading contributor to childhood morbidity. The leading viral pathogens associated with ARI in high-income settings are respiratory syncytial virus (RSV), parainfluenza virus, influenza virus and rhinovirus [8–10]. In 2019, RSV-alone was estimated globally to cause 3.6 million hospital admissions for ARI in children aged less than 5 years [11]. Preterm infants, and/or those of low birthweight (< 2500 g) as well as children with CLD are at greater risk of severe morbidity due to viral ARIs [12, 13]. Given these characteristics, collectively, NICU graduates are a particular high-risk group for ARI morbidity and subsequent health service utilisation.

Prevention measures, such as immunoprophylaxis with the RSV monoclonal antibody, palivizumab, are recommended to select groups of infants, including those born extremely preterm within the NICU to reduce severe infections caused by RSV, [14, 15] but we have reported low use in our setting [16]. Lung function trajectories are impaired in survivors of very preterm birth, [17] increasing the susceptibility to viral ARIs. However, the longer-term patterns of morbidity in all NICU graduates including those who are not preterm, remains unknown, particularly for respiratory infections occurring beyond infancy. With near-to-market RSV prevention measures aimed at all infants, and not only those preterm, [18] quantifying the long-term burden from ARI in NICU graduates would be useful to evaluate post-licensure effectiveness.

Our aim in this study is to describe health service utilisation patterns for ARI in a population cohort of all NICU graduates recorded on an administrative NICU database in terms of incidence rates of subsequent emergency department (ED) presentations and hospitalisations in the first 8 years of life. We describe ED presentations and hospitalisations by age group and ARI diagnosis, and further investigate ARI hospitalisations by selected at-risk groups within the NICU.

## Methods

### Study setting

Western Australia (WA) is the largest state in Australia, covering approximately 2.5 million square kilometres, and in 2016 had an estimated population of 2.5 million [19]. In 2013, 2.6% of all registered live births in Australia had a NICU admission; since 2004, this proportion has varied from 2.2% to 2.6% [1]. In WA there is only one tertiary neonatal directorate, with one NICU located at Princess Margaret Hospital for Children (now Perth Children’s Hospital) and the other at the only perinatal centre, King Edward Memorial Hospital for Women.

### Study population and data sources

We conducted a retrospective record linkage cohort study. Details of this study and description of the use and effectiveness of palivizumab are described elsewhere [16, 20]. Briefly, the cohort was defined as infants born in WA between 1 January 2002 and 31 December 2013 inclusive, and admitted to the NICU at either Princess Margaret Hospital for Children or King Edward Memorial Hospital after birth. Infants who died before they were discharged from NICU or hospital were excluded; hence our cohort was defined as NICU graduates. The WA NICU database was used to identify the study cohort which has full capture of NICU activity with no missing data on variables required for this analysis. Outcome data came from three databases with varying end dates depending on the time of data extraction prior to linkage. Mortality data was available from the Death Registry, with all deaths in the cohort, between January 2002 and December 2015, being sourced. Hospitalisations were identified from the Hospital Morbidity Data Collection (HMDC) between January 2002 and June 2015 and emergency department presentations were identified from the Emergency Department Data Collection (EDDC) between January 2002 and October 2015. The HMDC includes all hospital admissions and separations from public and private free-standing hospitals across WA and the EDDC contains information for emergency department activity in public and private hospitals across WA. Records from these datasets were probabilistically linked by the Western Australia Department of Health using a set of unique person identifiers and according to established best practice protocols for linking administrative data [21].

### Disease classification

Each hospitalisation in the linked dataset has a primary diagnosis code, and up to 21 additional diagnosis codes, classified using the International Statistical Classification of Diseases and Related Health Problems 10^th^ Revision, Australian modification (ICD-10-AM). Classification rules used to identify ARI diagnoses in the HMDC and EDDC were based on our prior research in this area [22, 23] and are listed in Supplementary Table 1. Each of primary and additional diagnosis codes were used in the hospital data to classify ARI. ED presentations are either coded with a single ICD-10-AM diagnosis, a symptom code, and/or a field where free text can be entered (see Supplementary Table 1 for search terms). Disease classifications for ARI were made using regular expressions by mapping: the ICD-10-AM codes against the diagnosis field(s), symptom codes (ED presentations only), and free text fields (ED presentations only). As in our previous analyses, symptom codes and free text fields in ED are only used if the ICD code is missing [23].

### Episodes of infection

Dates of admission and separation were available for the NICU and HMDC dataset and date of presentation was available for the EDDC dataset. As per our previous research using these datasets [24] any hospitalisation (or NICU stay) within 14 days of a previous hospitalisation were collapsed into a single ‘episode of infection’ (episode of care). Each morbidity record (HMDC or EDDC) was assessed separately and given a unique diagnosis classification. Given a single episode of infection may have multiple records from varied sources with numerous diagnosis classifications, the “overall ARI diagnosis” was determined by a hierarchical diagnosis that ranked diseases in order of clinical severity: whooping cough/pertussis, pneumonia, bronchiolitis, influenza, unspecified acute lower respiratory infection, bronchitis, URTI, and other ARI unrelated diagnosis. This hierarchal diagnosis approach follows our previously published work [22, 25].

### Time-at-Risk

Time-at-risk for each child during the study commenced 24 h following separation from their birth-related NICU episode of care and ended upon either their death or the end of the study period (December 31, 2015), whichever occurred first. Children were not classified as ‘at risk of infection’ if there were already in hospital.

### Statistical analysis

ARI incidence rates were calculated using the number of distinct episodes of care for each overall ARI diagnosis and the total time at risk and reported per 1,000 child-years. ARI incidence rates (for each diagnosis classification) were calculated (as above) separately by age in pre-specified age groups (0–5 months, 6–11 months, 12–23 months, 2–3 years, and 4–8 years) and year of hospitalisation (2002 to 2015). ARI hospitalisation rates (noted as hospital admission rates) were further examined between chosen at-risk groups which included gestational age and presence of CLD. Gestational age at birth was categorised into extremely preterm (< 28 weeks gestation), very preterm (28–32 weeks), moderate to late preterm (33–36 weeks) and term (≥ 37 weeks). CLD was defined using an indicator variable on the NICU database (for oxygen requirement after 36 weeks of postmenstrual age) which is the standardised definition used in NICUs within the Australian and New Zealand Neonatal Network [1] as well as all ICD-10 AM diagnosis codes in the hospitalisation dataset (P27.1). These groups were chosen as they represented criterion groups for palivizumab recommendations under extended guidelines from 2010 in the King Edward Memorial Hospital NICU [20].

Poisson regression was used to investigate the differences in rates of ARI hospital admission between gestational age groups and those with CLD, after adjusting for age at hospital admission. These models included an offset term defined as the natural log of the adjusted time at risk. Results are reported as incidence rate ratios (IRR) with 95% confidence intervals. As per data custodian requirements, individual cell sizes of less than 5 in the results tables have been suppressed. Ethics approvals were granted by the Western Australian Department of Health and Child Adolescent Health Service (RGS2503). Data access was approved by the Western Australian Data Linkage Branch and relevant data custodians. All data cleaning and analysis was completed with R version 3.4.4 with RStudio.

## Results

Our cohort consisted of 24,090 children born in WA between Jan 1, 2002 and Dec 12, 2013 who were admitted to NICU during their birth episode. Of these, 306 infants died before discharge. Subsequently, our analytical cohort consisted of 23,784 NICU graduates providing an overall time-at-risk of 177,367 child-years (with follow-up to 2015). Of this cohort, 12,657 (53.2%) were born preterm (< 37 weeks gestation at birth) with 3,606 (15.2% of the total cohort) being very preterm (28–32 weeks) and 1,049 (4.4% of the total cohort) being extremely preterm (< 28 weeks gestation). Of those extremely preterm, 688 (65.6%) were classified as having CLD and 230 (6.4%) of those very preterm were classified as having CLD. The presence of CLD was low in those who were moderate to late preterm (33–36 weeks gestation, 0.2%) or term (0.1%). Other general cohort characteristics are presented elsewhere [16, 20]. The length of NICU-associated stay from birth to first hospital discharge ranged from 2 to 1,410 days (median 27.5 days; inter-quartile range [IQR] 39 days).

**Fig. 1:**
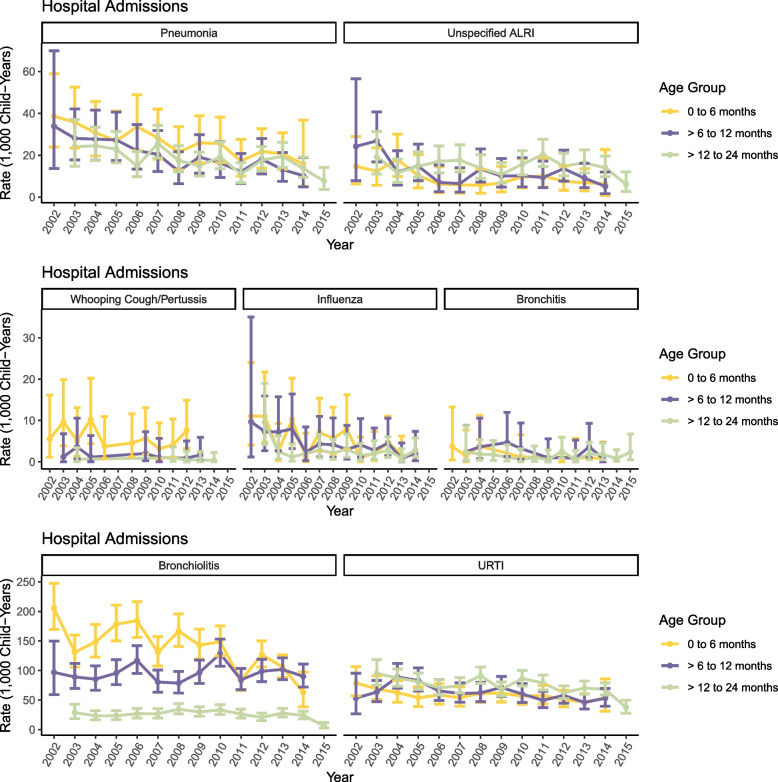
Annual rates of ARI hospital admissions from 2002 to 2015 by age at admission; 95% confidence intervals are indicated by error bars

From 2002 to 2015, the NICU graduate cohort had 47,443 subsequent hospital admissions and 123,900 subsequent ED presentations, across 57,515 episodes of care. Per individual, the median number of hospital admission episodes of care per NICU graduate was 2 (range 1 to 38; IQR 2 days) and the median number of emergency department presentations was 4 (range 1 to 212; IQR 6 days). ARI hospital admission rates for NICU graduates between 2002 and 2015 are provided in Table 1. The overall hospitalisation rate for infants aged up to 8 years was 71.4/1000 child-years. The highest rates overall were seen in children in the first 6 months of life (242.9/1000) which then declined with age. Rates were highest for bronchiolitis, especially in younger children with the rate of 139.4/1000 in those aged 0–5 months and 95.6/1000 in those aged 6–11 months (Table 1). Rates were also high for URTI, especially in the second year of life (74.2/1000 for those aged 12–23 months). Annual rates of ARI hospital admission between 2002 and 2015 are shown in Fig. 1. ARI hospital admissions declined over this interval, more markedly in infants aged less than 12 months where, for example, rates were 165/1000 (95% CI: 114/1000 to 230/1000) in 2002 for those aged 6–11 months which declined to 107/1000 (95% CI: 87.8/1000 to 130/1000) in 2014; Fig. 1).

**Table 1:** Number and incidence rates of hospital admissions by age and diagnosis in infants attending NICU, 2002–2015

Hierarchical Diagnosis	0–5 months		6–11 months		12–23 months		2–3 years		4–8 years		Overall	
	**n**	**Rate**^a^ ***(95%CI)***	**n**	**Rate**^a^ ***(95%CI)***	**n**	**Rate**^a^ ***(95%CI)***	**n**	**Rate**^a^ ***(95%CI)***	**n**	**Rate**^a^ ***(95%CI)***	**n**	**Rate**^a^ ***(95%CI)***
Whooping Cough	49	4.6 *(3.4, 6.0)*	11	0.9 *(0.5, 1.7)*	6	0.3 *(0.1, 0.6)*	8	0.2 *(0.1, 0.4)*	< 5	0.0 *(0.0, 0.1)*	75	0.4 *(0.3, 0.5)*
Pneumonia	279	26.0 *(23.1, 29.3)*	221	18.7 *(16.3, 21.3)*	418	17.6 *(16.0, 19.4)*	415	9.8 *(8.9, 10.8)*	190	3.3 *(2.8, 3.8)*	1523	8.6 *(8.2, 9.0)*
Bronchiolitis	1493	139.4 *(132.4, 146.6)*	1132	95.6 *(90.1, 101.4)*	614	25.9 *(23.9, 28.0)*	89	2.1 *(1.7, 2.6)*	12	0.2 *(0.1, 0.4)*	3340	18.8 *(18.2, 19.5)*
Influenza	53	4.9 *(3.7, 6.5)*	49	4.1 *(3.1, 5.5)*	51	2.2 *(1.6, 2.8)*	61	1.4 *(1.1, 1.9)*	25	0.4 *(0.3, 0.6)*	239	1.3 *(1.2, 1.5)*
Unspecified ALRI	97	9.1 *(7.3, 11.0)*	136	11.5 *(9.6, 13.6)*	352	14.8 *(13.3, 16.5)*	340	8.0 *(7.2, 8.9)*	139	2.4 *(2.0, 2.8)*	1064	6.0 *(5.6, 6.4)*
Bronchitis	10	0.9 *(0.4, 1.7)*	19	1.6 *(1.0, 2.5)*	33	1.4 *(1.0, 2.0)*	22	0.5 *(0.3, 0.8)*	7	0.1 *(0.0, 0.2)*	91	0.5 *(0.4, 0.6)*
URTI	621	58.0 *(53.5, 62.7)*	738	62.3 *(57.9, 67.0)*	1760	74.2 *(70.8, 77.8)*	2047	48.3 *(46.3, 50.5)*	1158	19.9 *(18.8, 21.1)*	6324	35.7 *(34.8, 36.5)*
Overall ARI	2602	242.9 *(233.6, 252.4)*	2306	194.8 *(186.9, 202.9)*	3234	136.4 *(131.7, 141.1)*	2982	70.4 *(67.9, 73.0)*	1532	26.3 *(25.0, 27.7)*	12,656	71.4 *(70.1, 72.6)*

*Abbreviations*: *ALRI* acute lower respiratory infection, *ARI* acute respiratory infections, *n* number, *NICU* neonatal intensive care unit, *URTI* upper respiratory tract infection

^a^Rate per 1000 child-years

**Fig. 2:**
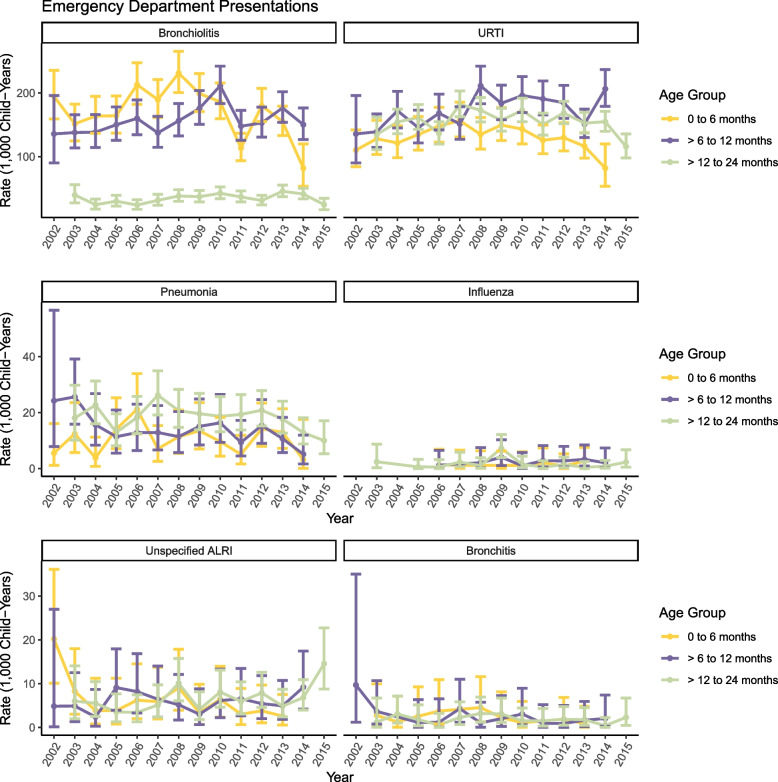
Annual rates of ARI emergency department presentations from 2002 to 2015 by age at presentation; 95% confidence intervals are indicated by error bars. NOTE: Annual presentation rates for whooping cough not shown due to small numbers

The frequency of ED presentations in NICU graduates from 2002–2015 by overall ARI diagnosis classification and age, was similar to that observed for hospital admissions (Table 2). Overall, the ARI ED presentation rate was 114.0/1000 and was highest in those aged 0–5 months (337.6/1000) and 6–11 months (366.0/1000) and then declined with age. Bronchiolitis and URTI were the most common diagnoses in infants aged less than 12 months and URTI remained the most common diagnosis in children beyond 2 years of age (e.g., 92.7/1000 in those aged 2–3 years and 31.1/1000 in those aged 4–8 years; Table 2). Annual rates of ED presentations for ARI between 2002 and 2015 are shown in Fig. 2. Unlike hospital admissions, overall ED presentation rates did not change over time, except for in those aged 0–5 months, where after rates peaked at 258/1000 (95% CI: 226/1000 to 294/1000) in 2008, rates declined to 85/1000 (95% CI: 59/1000 to 124/1000) in 2014 (Fig. 2).

**Table 2:** Number and incidence rates of emergency department presentations by age and diagnosis in infants attending NICU, 2002–2015

Hierarchical Diagnosis	0–5 months		6–11 months		12–23 months		2–3 years		4–8 years		Overall	
	**n**	**Rate**^a^ ***(95%CI)***	**n**	**Rate**^a^ ***(95%CI)***	**n**	**Rate**^a^ ***(95%CI)***	**n**	**Rate**^a^ ***(95%CI)***	**n**	**Rate**^a^ ***(95%CI)***	**n**	**Rate**^a^ ***(95%CI)***
Whooping Cough	46	4.3 *(3.1, 5.7)*	15	1.3 *(0.7, 2.1)*	12	0.5 *(0.3, 0.9)*	14	0.3 *(0.2, 0.6)*	10	0.2 *(0.1, 0.3)*	97	0.5 *(0.4, 0.7)*
Pneumonia	119	11.1 *(9.2, 13.3)*	170	14.4 *(12.3, 16.7)*	456	19.2 *(17.5, 21.1)*	484	11.4 *(10.4, 12.5)*	218	3.7 *(3.3, 4.3)*	1447	8.2 *(7.7, 8.6)*
Bronchiolitis	1933	180.4 *(172.5, 188.7)*	1937	163.6 *(156.4, 171.1)*	847	35.7 *(33.3, 38.2)*	116	2.7 *(2.3, 3.3)*	< 5	0.0 *(0.0, 0.1)*	4835	27.3 *(26.5, 28.0)*
Influenza	11	1.0 *(0.5, 1.8)*	20	1.7 *(1.0, 2.6)*	37	1.6 *(1.1, 2.2)*	47	1.1 *(0.8, 1.5)*	32	0.6 *(0.4, 0.8)*	147	0.8 *(0.7, 1.0)*
Unspecified ALRI	61	5.7 *(4.4, 7.3)*	70	5.9 *(4.6, 7.5)*	156	6.6 *(5.6, 7.7)*	156	3.7 *(3.1, 4.3)*	69	1.2 *(0.9, 1.5)*	512	2.9 *(2.6, 3.1)*
Bronchitis	19	1.8 *(1.1, 2.8)*	23	1.9 *(1.2, 2.9)*	46	1.9 *(1.4, 2.6)*	40	0.9 *(0.7, 1.3)*	13	0.2 *(0.1, 0.4)*	141	0.8 *(0.7, 0.9)*
URTI	1428	133.3 *(126.5, 140.4)*	2097	177.2 *(169.7, 184.9)*	3775	159.2 *(154.1, 164.3)*	3927	92.7 *(89.8, 95.7)*	1807	31.1 *(29.7, 32.5)*	13,034	73.5 *(72.2, 74.8)*
Overall ARI	3617	337.6 *(326.7, 348.8)*	4332	366.0 *(355.2, 377.1)*	5329	224.7 *(218.7, 230.8)*	4784	113.0 *(109.8, 116.2)*	2151	37.0 *(35.4, 38.6)*	20,213	114.0 *(112.4, 115.5)*

*Abbreviations*: *ALRI* acute lower respiratory infections, *ARI* acute respiratory infection, *n* number, *NICU* neonatal intensive care unit, *URTI* upper respiratory tract infection

^a^Rate per 1000 child-years

We assessed ARI hospitalisation rates within gestational age groups and by CLD status for those who were classified as extremely or very preterm (Table 3). ARI admission rates were highest in those born extremely preterm (< 28 weeks gestation at birth) with CLD and these higher rates persisted into early childhood. The ARI rate for 0–5 months for those extremely preterm with CLD was 1149.6/1000 compared with 181.9/1000 for those born at term. The group with the next highest ARI rates across all ages were those born very preterm (28–32 weeks) with CLD (e.g., 731.3/1000 for those 0–5 months) and this was higher than those extremely preterm with no CLD (e.g., 581.8/1000 for those 0–5 months, Table 3). ARI rates declined with age across all groups, however the discrepancy in rates between preterm groups with and without CLD persisted into early childhood. For example, at age 4–8 years, ARI rates were 60.7/1000 in those born extremely preterm with CLD and 44.9/1000 in those very preterm with CLD which was still 2.6 and 1.9 times higher, respectively, than ARI rates at age 4–8 years in those born at term (23.6/1000; Table 3). We conducted Poisson regression analysis to formally compare ARI hospitalisation rates between age at admission, and levels of preterm birth shown in Table 4 and presence of CLD in Table 5. After adjusting for age at subsequent hospital admission, on average, children born extremely preterm experienced ARI-related subsequent hospital admissions 6.51 times higher (95% CI: 6.01–7.04) than children born at term (Table 4). The largest discrepancy was seen with bronchiolitis. After adjusting for age at hospitalisation, on average, the IRR for a child of any gestational age with CLD being hospitalised with ARI, compared to a child without CLD, was 5.04 (95% CI: 4.69 to 5.41) with the largest discrepancy seen with bronchiolitis and unspecified ALRI (Table 5).

**Table 3:** Number and incidence rates of ARI hospital admission by gestational age and chronic lung disease at different ages of admission

Group	0–5 months		6–11 months		12–23 months		2–3 years		4–8 years		Overall	
	**n**	**Rate**^a^ ***(95%CI)***	**n**	**Rate**^a^ ***(95%CI)***	**n**	**Rate**^a^ ***(95%CI)***	**n**	**Rate**^a^ ***(95%CI)***	**n**	**Rate**^a^ ***(95%CI)***	**n**	**Rate**^a^ ***(95%CI)***
< 28w + CLD	157	1149.6 *(976.8, 1344.2)*	227	686.7 *(600.3,782.1)*	311	459.5 *(409.8, 513.5)*	265	215.4 *(190.2, 242.9)*	102	60.7 *(49.5, 73.7)*	1062	213.4 *(200.7, 226.6)*
< 28w no CLD	61	581.8 *(445.1, 747.4)*	79	441.9 *(346.9, 550.8)*	85	236.8 *(189.2, 292.9)*	68	102.5 *(79.6, 129.9)*	32	31.9 *(21.8, 45.0)*	325	119.0 *(106.4, 132.7)*
28–32w + CLD	49	731.3 *(541.0, 966.8)*	57	523.0 *(396.1, 677.6)*	59	270.0 *(205.5, 348.3)*	65	170.3 *(131.4, 217.0)*	24	44.9 *(28.8, 66.9)*	254	152.4 *(134.2, 172.3)*
28–32w no CLD	372	433.4 *(390.5, 479.8)*	317	288.8 *(257.8, 322.4)*	400	181.9 *(164.5, 200.6)*	344	85.7 *(76.9, 95.2)*	185	32.5 *(28.0, 37.5)*	1618	97.1 *(92.4, 101.9)*
33–36w	997	235.3 *(221.0, 250.4)*	796	174.2 *(162.3, 186.7)*	1168	127.6 *(120.4, 135.1)*	1048	63.8 *(60.0, 67.8)*	569	24.7 *(22.7, 26.9)*	4578	66.3 *(64.4, 68.2)*
≥ 37w	966	181.9 *(170.6, 193.8)*	830	149.6 *(139.6, 160.1)*	1211	109.0 *(103.0, 115.3)*	1192	60.7 *(57.3, 64.2)*	620	23.6 *(21.8, 25.6)*	4819	58.6 *(57.0, 60.3)*

Abbreviations: CLD, chronic lung disease; n, number; NICU, neonatal intensive care unit; w, weeks gestation

^a^Rate per 1000 child-years

**Table 4:** Incidence rate ratios by admission and gestational age from Poisson regression by ARI diagnosis

Risk Group	Incidence rate ratio (95% confidence interval)						
	**Overall**	**Whooping Cough**	**Pneumonia**	**Bronchiolitis**	**Influenza**	**Unspecified ALRI**	**Bronchitis**
Age at admission							
0–5 months	Reference	Reference	Reference	Reference	Reference	Reference	Reference
6–11 months	0.670 *** (0.627, 0.716)	0.192 *** (0.094, 0.355)	0.672 *** (0.562, 0.801)	0.635 *** (0.588, 0.686)	0.799 (0.540, 1.179)	1.200 (0.926, 1.561)	1.741 (0.827, 3.900)
12–23 months	0.314 *** (0.293, 0.336)	0.061 *** (0.024, 0.133)	0.633 *** (0.544, 0.737)	0.172 *** (0.156, 0.188)	0.414 *** (0.282, 0.610)	1.547 *** (1.241, 1.948)	1.347 (0.684, 2.897)
2–3 years	0.107 *** (0.099, 0.116)	0.039 *** (0.017, 0.077)	0.350 *** (0.301, 0.408)	0.014 *** (0.011, 0.017)	0.277 *** (0.191, 0.401)	0.834 (0.669, 1.051)	0.501 (0.242, 1.112)
4–8 years	0.036 *** (0.032, 0.040)	0.008 *** (0.000, 0.037)	0.116 *** (0.096, 0.139)	0.001 *** (0.001, 0.002)	0.082 *** (0.050, 0.131)	0.247 *** (0.191, 0.321)	0.130 *** (0.047, 0.339)
Gestational age							
< 28w	6.510 *** (6.013, 7.041)	5.246 *** (2.080, 11.599)	4.999 *** (4.272, 5.831)	6.783 *** (6.067, 7.572)	3.922 *** (2.492, 5.977)	4.082 *** (3.389, 4.892)	4.610 ** (1.683, 10.809)
28–32w	2.637*** (2.449, 2.837)	1.944 (0.817, 4.147)	2.106*** (1.805, 2.449)	3.296*** (2.983, 3.641)	2.290*** (1.555, 3.316)	1.521*** (1.253, 1.835)	2.711*** (1.367, 5.173)
33–36w	1.351*** (1.273, 1.434)	1.684* (1.009, 2.850	1.169* (1.036, 1.319)	1.563*** (1.438, 1.700)	1.299 (0.963, 1.753)	0.960 (0.832, 1.106)	2.366*** (1.462, 3.930)
≥ 37w	Reference	Reference	Reference	Reference	Reference	Reference	Reference

*Abbreviations*: *ALRI* acute lower respiratory infection, *ARI* acute respiratory infection, *w* weeks gestation

^***^p < 0.001; ** *p* < 0.01; * *p* < 0.05

**Table 5:** Incidence rate ratios by admission age and CLD from Poisson regression by ARI diagnosis

Risk Group	Incidence rate ratio (95% confidence interval)						
	**Overall**	**Whooping Cough**	**Pneumonia**	**Bronchiolitis**	**Influenza**	**Unspecified ALRI**	**Bronchitis**
Age at admission							
0–5 months	Reference	Reference	Reference	Reference	Reference	Reference	Reference
6–11 months	0.670 *** (0.627, 0.716)	0.190 *** (0.094, 0.352)	0.675 *** (0.565, 0.805)	0.641 *** (0.593, 0.693)	0.800 (0.541, 1.180)	1.185 (0.914, 1.541)	1.636 (0.776, 3.667)
12–23 months	0.314 *** (0.293, 0.336)	0.052 *** (0.020, 0.111)	0.636 *** (0.546, 0.740)	0.173 *** (0.157, 0.190)	0.415 *** (0.282, 0.610)	1.526 *** (1.224, 1.921)	1.416 (0.723, 3.032)
2–3 years	0.111 *** (0.103, 0.120)	0.038 *** (0.017, 0.077)	0.353 *** (0.303, 0.411)	0.014 *** (0.011, 0.017)	0.278 *** (0.192, 0.402)	0.825 (0.661, 1.039)	0.528 (0.256, 1.167)
4–8 years	0.034 *** (0.031, 0.038)	0.004 *** (0.000, 0.019)	0.118 *** (0.098, 0.141)	0.001 *** (0.001, 0.002)	0.083 *** (0.051, 0.132)	0.246 *** (0.190, 0.319)	0.142 *** (0.052, 0.371)
Chronic Lung Disease							
No CLD	Reference	Reference	Reference	Reference	Reference	Reference	Reference
CLD	5.040 *** (4.694, 5.405)	5.112 *** (2.364, 9.778)	4.629 *** (4.004, 5.326)	5.123 *** (4.635, 5.650)	3.662 *** (2.414, 5.340)	5.178 *** (4.396, 6.066)	3.928 *** (1.968, 7.097)

*Abbreviations*: *ALRI* acute lower respiratory infection, *ARI* acute respiratory infection, *CLD* chronic lung disease

^***^p < 0.001; ** *p* < 0.01; * *p* < 0.05

## Discussion

From a population cohort of infants being discharged from NICU, we have successfully described the health service utilisation and the resulting ongoing burden of ARI with overall rates of ARI in children up to age 8 years of 71/1000 child-years for hospital admission and 114/1000 child-years for ED presentations. Although ARI secondary care rates in NICU graduates were highest in the first year of life, the burden of ARI persisted into early childhood, even in those who were not in our pre-determined at-risk groups such as those born preterm, with or without CLD. However, within the NICU cohort, extreme preterm infants or those with CLD were 5–6 times more likely to be subsequently admitted to hospital for an ARI compared to term-born NICU graduates or those without CLD.

A previous population-based birth cohort of 337,909 births in WA between 2001 and 2012 reported ARI rates in infants aged less than 12 months at 43.7/1000 [26]. Here, we report rates of ARI in NICU graduates are approximately 4.5–5.5 times higher, representing a significant disproportionate burden. These high rates of ARI hospitalisation and ED presentation will likely represent a significant health service cost and associated societal and economic burden that warrants attention, especially as rates of preterm birth and CLD are not declining. Bronchiolitis had the highest rate of all ARI disease categories, a finding consistent with numerous studies. Respiratory syncytial virus (RSV) is the major pathogen associated with bronchiolitis and pneumonia in children [9, 27] and there is renewed interest in global RSV prevention through maternal vaccination strategies and single dose longer-acting monoclonal antibodies; the latter of which has been shown in clinical trials to reduce medically-attended RSV ARI in preterm infants by 70% [28]. The licensure and introduction of such promising therapeutics is therefore likely to be a benefit in reducing the ARI burden in NICU graduates and associated studies addressing awareness and acceptability of future RSV prevention measures studies are now needed. Single-dose long-acting monoclonal antibodies are close to market for RSV, [18] and while they are expected to reduce ARI rates in young infants, their impact on long-term ARI morbidity is unknown. Our data presented here provide robust baseline data that can be compared in post-licensure effectiveness studies once such long-acting monoclonal antibodies are available in our population.

Similar to previous total population birth cohort analyses, [29] we report an ongoing burden of ARI in those born preterm and with CLD up to age 8 years in our NICU graduating cohort. There is now a large body of evidence supporting the detrimental role of early life ARI on poor respiratory health outcomes through life, including low lung function, [30] the early origins of chronic obstructive pulmonary disorder (COPD) [31] and asthma diagnosis. Indeed, a popular paradigm postulates that recurrent respiratory viral infections at critical time periods of immune and lung development in infancy and childhood, coupled with allergic sensitisation, are associated with the development of asthma [32].

It is likely that the negative impacts of early life ARI are further amplified for survivors of preterm birth, who have immature lungs and other adverse early life exposures such as invasive respiratory support in the NICU. While no studies have explicitly looked at the link between early life ARI and later lung health outcomes in survivors of preterm birth, recent studies show that the respiratory health burden after preterm birth is high. Survivors of preterm birth are up to five times more likely to develop childhood wheeze or “asthma” than their term counterparts, [33] and have reduced lung function during childhood [17] that is progressively diverging further from the “normal” trajectory over time [34, 35].

The mechanisms underlying increased risk of more frequent and/or severe ARI in the years following preterm birth are currently unclear. However, emerging data suggest a multifactorial foundation. Preterm infants have an immature cellular innate and adaptive immune system at birth, as well as lower immunoglobulin levels since maternal transfer normally occurs in the third trimester of pregnancy [36, 37]. Further, the microbiome helps shape the innate immune system and contributes to effective barrier function, but infants born preterm have low microbial diversity and altered dominant microbial species [38]. Altered microbiota composition persists to young-adulthood and is correlated with reduced lung function in this population [39]. Our findings of increased severe ARI through childhood in preterm babies from NICU and emerging evidence from these research areas, suggest that those born preterm have reduced ability to detect and effectively eliminate respiratory pathogens during early life and beyond; highlighting the importance of further work in this area. However, as 47% of our NICU cohort were born at term, the ongoing burden of ARI is not just limited to preterm birth and measures to prevent respiratory pathogens in early life need to be applicable to all infants.

The main strength of our study is in the availability and use of population-based datasets, with complete perinatal and demographic information to form the study cohort. This allowed us to document age-specific ARI incidence rates up to age 8 years, where other studies have only assessed frequency of re-hospitalisation following NICU discharge up to age 2 or 3 years [40, 41]. The higher frequency reported in these studies, along with our findings here, suggests this is indicative of a true higher disease burden in NICU graduates, and not due to health seeking practices in our jurisdiction.

Our observational study is not without limitations. First, there are inherent difficulties with using ICD codes to distinguish different respiratory infection presentations, as highlighted in our previous analyses of administrative health data [9]. However, we have used validated sets of diagnosis codes used in our prior research to classify each ARI diagnosis. While these outcomes represent clinical diagnoses and not virus-specific outcomes, we feel these are still useful as baseline data for future immunisation strategies against RSV, considering that in our population, the most common aetiological agent for bronchiolitis and pneumonia hospitalisations is RSV. Second, we chose a priori to examine age-specific ARI rates within two key risk groups in NICU (children born preterm and those with CLD) based on extended guidelines for palivizumab recommendations in our jurisdiction to provide further background for assessing palivizumab effectiveness [20]. However, we acknowledge there are other risk groups that necessitate admission to NICU. Finally, there are other socio-demographic factors and population subgroups that we did not assess ARI rates for in the cohort of NICU graduates including Aboriginal and/or Torres Strait Islander children and children from lower socio-economic backgrounds.

## Conclusions

Our study highlights the on-going impact of NICU admittance, as well as preterm birth and its complications (CLD) on respiratory health, and well-designed studies examining the lifelong impact of early ARI on later lung health are urgently needed in this population. Minimising early life respiratory infections in NICU graduates through parental education and awareness on the importance of on-time current infant vaccines targeting respiratory infections and future RSV monoclonal antibodies as well as non-pharmaceutical prevention measures such as increased hand hygiene, social distancing from others when symptomatic, should also be an urgent priority in this high-risk population.

## Supplementary Information

**Additional file 1: Supplementary Table 1.** Diagnosis, symptom and discharge codes for acute respiratory infection in hospitalisation and emergency department data.

## Data Availability

This study used individual record data. Data are not available to the public but can be required from the Western Australian Department of Health. Code for data analyses can be made available by request from HCM.

## Abbreviations

ARI: Acute respiratory infection
CLD: Chronic lung disease
ED: Emergency department
EDDC: Emergency department data collection
HMDC: Hospital morbidity data collection
ICD: International Classification of Diseases
IRR: Incidence rate ratio
NICU: Neonatal intensive care unit
RSV: Respiratory syncytial virus
URTI: Upper respiratory tract infection
WA: Western Australia
