# Supplementary material for: Health service utilisation for acute respiratory infections in infants graduating from the neonatal intensive care unit: a population-based cohort study
Source: BMC Pediatr. 2023 Jul 1;23:335. doi: 10.1186/s12887-023-04152-5 (PMC10314380; doi:10.1186/s12887-023-04152-5)
Supplement: Supplementary file 1 — Additional file 1: Supplementary Table 1. Diagnosis, symptom and discharge codes for acute respiratory infection in hospitalisation and emergency department data. [file 12887_2023_4152_MOESM1_ESM.docx]

Health service utilisation for acute respiratory infections in infants graduating from the neonatal intensive care unit: a population-based cohort study

**Supplementary Table 1.** Diagnosis, symptom and discharge codes for acute respiratory infection in hospitalisation and emergency department data

| **Diagnosis category** | **International Classification of Diseases version 10, Australian Modification code**  **(Hospital)** | **Symptom code*** | **Presenting complaint/discharge diagnosis (free text)*** |
| --- | --- | --- | --- |
| **Acute lower Respiratory Infection ALRI)** | |  |  |
| Whooping Cough | A37 | SNJ Pertussis/whooping cough | Pertussis; whooping cough; post-tussive vomiting |
| Pneumonia | J12–J18, B01.2, B05.2, B37.1, B59 | SQJ Pneumonia | Pneumonia |
| Bronchiolitis | J21 | SNB00 Bronchiolitis | Bronchiolitis |
| Influenza | J09-J11 | AAV Flu Like Symptoms | Influenza; flu; flu-like symptoms |
| Unspecified acute lower respiratory infection | J22 | SQD Chest infection | Unspecified acute lower respiratory infection; LRTI; lower respiratory tract infection; chest infection |
| Bronchitis | J20, J40 | SQC Bronchitis | Bronchitis |
| **Upper Respiratory infection (URTI)** | |  |  |
| URTI | J00-J06, R06.1, H65-67, H70-75, H83, H90, H92, H95, R56.0^, R56.8^, R06.2^, R05^, B34^, R50^ | CG Stridor; SNG Febrile convulsion; CH Wheeze; CC Cough; S2B Pyrexia of unknown origin; VP Pyrexia of unknown origin; VD Fever; PG Febrile; AAU Fever; FE Nasal discharge | Croup; laryngotracheobronchitis; barking cough; stridor; febrile convulsion; convulsion; wheeze; wheezing; cough; crackles; viral respiratory infection; viral respiratory tract infection; fever; pyrexia; febrile; high temperature; otitis media; upper respiratory tract infection; URTI; nasopharyngitis; sinusitis; pharyngitis; laryngitis; tracheitis; epiglottitis; rhinitis; runny nose, nasal discharge |

*Emergency Department Data only

^Due to only one available code available in ED data, additional codes were used to identify URTI in ED
